# Antimicrobial Coating of Surgical Meshes by Laser-Induced Nanocarbon Synthesis and Transfer

**DOI:** 10.1021/acsapm.6c00879

**Published:** 2026-06-08

**Authors:** Diala Bani Mustafa, Josiane Semaan, Soumalya Ghosh, Nathan Carney, Tagbo H. R. Niepa, Rui Liang, Mostafa Bedewy

**Affiliations:** † Department of Mechanical Engineering and Materials Science, 6614 University of Pittsburgh, Pittsburgh, Pennsylvania 15261, United States; ‡ Department of Anesthesiology, 2647 The Ohio State University, Columbus, Ohio 43210, United States; § Department of Chemical Engineering, department of Biomedical Engineering, 6612 Carnegie Mellon University, Pittsburgh, Pennsylvania 15213, United States; ∥ Department of Chemical and Petroleum Engineering, 6614 University of Pittsburgh, Pittsburgh, Pennsylvania 15261, United States; ⊥ Department of Industrial Engineering, 6614 University of Pittsburgh, Pittsburgh, Pennsylvania 15261, United States

**Keywords:** graphene, antibacterial surfaces, conformal coatings, laser-induced graphene, superhydrophobic

## Abstract

Conformal coatings on polypropylene (PP) surgical meshes remain challenging because filaments, knots, and junctions create shadowing and PP is thermally sensitive. We introduce laser-induced nanocarbon synthesis and transfer (LINCSAT), a one-step toner-assisted process that deposits porous sp^2^-rich carbon from a donor precursor onto PP without melting. Electron microscopy and Raman spectroscopy confirm continuous nanocarbon coverage across 3D mesh filaments and knots. 3D profilometry reveals a coating thickness up to ≈40 μm and enables antibacterial mapping versus local thickness. *Escherichia coli* live/dead assays show a nonmonotonic response, with ≈15 μm coatings minimizing viability. Overall, LINCSAT enables programmable antibacterial coatings on 3D thermoplastics.

## Graphical abstract

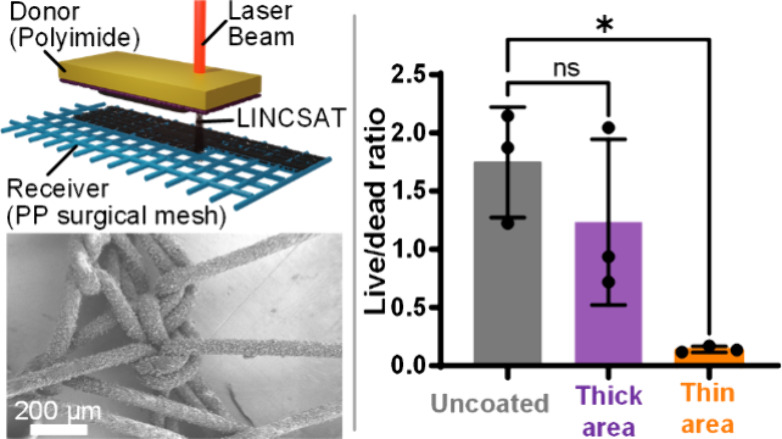

Polypropylene (PP) is widely used in surgical meshes because it offers mechanical robustness and favorable biocompatibility.^1,2^ However, PP can be susceptible to bacterial contamination, such as with *Escherichia coli* (*E. coli*) and *Staphylococcus aureus* (*S. aureus*) which is associated with infection-driven complications that can necessitate revision or removal.^3,4^ PP is low-service temperature polymer with intrinsic hydrophobicity, low surface energy, and chemical inertness, making conformal functional coatings difficult, particularly on open 3D architectures where filaments, knots, and junctions introduce shadowing and complex local topology.^5,6^ Conventional approaches such as collagen coatings, metallic/oxide layers, and antibiotic eluting systems often face trade-offs among coating uniformity, mechanical integrity, and sustained antibacterial function.^5,7^ Low temperature plasma activation is commonly used to increase PP surface energy and introduce polar functionalities that promote wetting and adhesion of coatings.^8^ Recent work on oxidative and plasma-based functionalization of PP meshes confirms that such treatments can enhance integration and provide anchoring sites for polymer, hydrogel, or inorganic coatings, but also highlights persistent challenges in achieving continuous, mechanically robust antimicrobial layers.^8,9^

3D porous graphene and related spiky nanocarbons are promising mesh-coating materials because they can provide chemically stable, mechanically resilient surfaces and have been reported to exhibit antibacterial activity while minimally altering bulk device mechanics.^10^ Laser-induced graphene (LIG) is a maskless, single-step laser conversion process that transforms carbon-rich solid precursors into porous, graphene-like networks whose morphology and chemistry depend on precursor composition and laser parameters.^11–13^ LIG have shown antimicrobial and antifouling performance against *E. coli* and other bacteria with outcomes that vary with surface functional groups and electroactivity under applied bias.^10,14^ However, LIG requires a graphitizable polymer precursor.^13,15^ In contrast, PP is nongraphitizing and thermally sensitive, and direct laser irradiation typically leads to melting/ablation rather than formation of sp^2^-rich carbon.^16^

In laser-induced nanocarbon synthesis and transfer (LINCSAT), we enable nanocarbon patterning on PP by decoupling the carbon precursor from the receiver: a graphitizable donor (polyimide, PI) is positioned above the PP substrate across a controlled gap (δ), as schematized in Figure 1a. To enhance optical coupling at near-infrared wavelengths, the donor underside is coated with a printed magnetic ink character recognition (MICR) toner for improved absorptance and localized heating.^17^ In our process, laser exposure converts the PI donor into nanostructured carbon, and the resulting rapid ejection transfers this material across the gap onto the receiver. The use of MICR is crucial as it greatly reduces the power required, thereby limiting thermal loading on low-service temperature polymers such as PP. Systematic tuning of process parameters controls the deposited LINCSAT coating thickness, morphology, wetting behavior, and antimicrobial properties.

**Figure 1:**
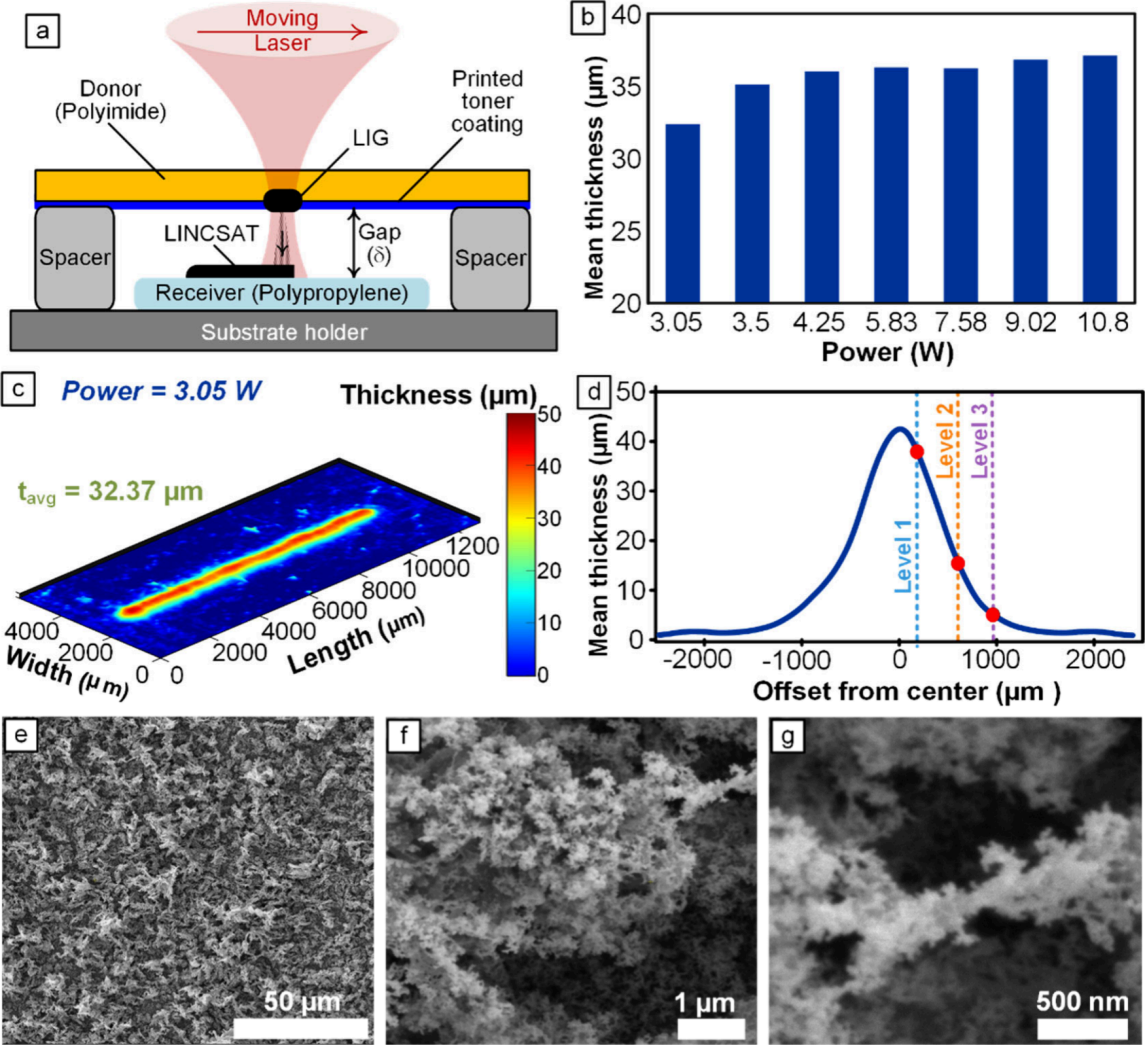
Process–structure relationships for toner-assisted LINCSAT coatings on polypropylene (PP) blocks. (a) Schematics of the LINCSAT process. (b) Mean coating thickness $t_{\mathrm{avg}}$ of single-pass LINCSAT lines as a function of laser power (the lowest power condition, 3.05 W, was selected for subsequent coating fabrication and antibacterial assays). (c) 3D optical profilometry map of a representative line produced at 3.05 W showing a continuous ridge along the scan direction and the lateral thickness distribution. (d) Mean thickness profile (averaged across the line width) used to define three characteristic thickness levels for subsequent antibacterial mapping. (e–g) Representative SEM images acquired from the ridge crest region (level 1) of a LINCSAT line produced at 3.05 W, showing the porous, spiky morphology at different magnifications.

The direct-write nature of LINCSAT enables controlled spatial variations in coating thickness on PP, allowing directly testing the thickness-dependence of bacterial attachment and viability under fixed assay conditions. Across the tested range, antibacterial performance exhibits a nonmonotonic dependence on coating thickness: an intermediate coating thickness minimizes the live/dead under the same assay conditions, whereas very thin and very thick regions show weaker efficacy. These results identify coating thickness as an explicit design parameter for antibacterial PP coatings and demonstrate that LINCSAT enables nanocarbon patterning on difficult-to-coat meshes.

We established the processing window on flat PP blocks using 3D optical profilometry, scanning electron microscopy (SEM), Raman spectroscopy, and energy-dispersive X-ray spectroscopy (EDS), as shown in Figures 1 and S1–S5. Single-pass LINCSAT lines were written under fixed scan conditions (80 mm/s speed, 200 ns pulse duration, and 400 kHz repetition frequency) and a donor-receiver gap of 1 mm while varying the 1064 nm laser power from 3.05 to 10.8 W. The mean ridge thickness ( $t_{\mathrm{avg}})$ , extracted from 3D maps remained within 30–38 μm (Figure 1b), indicating that the peak thickness varied modestly above a threshold power. A representative 3D thickness map at 3.05 W (Figure 1c) shows a continuous ridge with a central peak and laterally decaying shoulders across the line width. Macroscopic optical images (Figure S1) corroborate this behavior, higher powers darken and broaden the lines but do not produce damage, melting, or ablation of the PP substrate for these low power values. The profile of mean thickness as a function of offset from the centerline (Figure 1d) exhibits a peak near the center line followed by a monotonic pronounced decrease toward the edges. This gradient, even at fixed laser power, suggests that the explosive transport and deposition of nanocarbon across the donor-receiver gap are governed by plume divergence,^18,19^ rather than by simple local heating at the receiver surface in laser-induced forward-transfer type process.^20^ We single-out three thickness levels for quantitative analysis: peak crest (Level 1), intermediate shoulder (Level 2), and thinner outer region (Level 3). These levels are used for spatially mapping *E. coli* viability.

Raman spectroscopy confirms graphene-like, sp^2^-enriched carbon across the tested power range and across the three coating-thickness levels used for antibacterial mapping (Figures S2 and S4). All spectra show D and G bands near 1350 and 1580 cm^–1^ and a broad 2D feature near 2700 cm^–1^, consistent with turbostratic/nanocrystalline carbon reported in LIG systems.^13,15,21^ No systematic shift in band positions or *I*_D_/*I*_G_ ratios was observed with power over this window. Likewise, the spectra acquired from Level 1, Level 2, and Level 3 did not show obvious level-dependent changes in the D and G band features. These results suggest comparable structure and crystallinity beyond a threshold power for material jetting in LINCSAT and indicate that the lateral coating-thickness gradient is primarily associated with coating buildup rather than a detectable change in graphitic carbon structure. This robustness is a key advantage and is consistent with prior observations of kinetically controlled phenomena in LIG.^21,22^ SEM imaging supports this interpretation by showing that the ridge crest, corresponding to Level 1, consists of a porous network of branched and pointed nanocarbon features with abundant submicron edges and asperities (Figure 1e-g). Additional SEM images acquired across the same lateral gradient show that this porous morphology is retained from Level 1 to Level 3, although the apparent coating buildup decreases from the crest to the outer region (Figure S5). Such hierarchical roughness of LINCSAT with dense asperities is consistent with LIG and related nanocarbons.^11,14,21^ Combined with surface oxygen, these accessible high-surface-area structures may contribute to antibacterial activity through a combination of nanoscale topography, local membrane stress, and surface chemistry, consistent with prior reports on antibacterial surfaces.^10,14,17,22,23^ The present study does not isolate a single dominant mechanism.

Elemental mapping by EDS (Figure S3) shows the uniform dominant C signal with a weak and less uniform O signal in the LINCSAT coating. Notably, metallic inclusions are clearly visible with large micron-scale spherical Al particles and smaller clusters of Fe. The Fe inclusions are consistent with toner-derived iron oxide nanoparticles that survive the laser exposure and co-deposit with carbon, from the MICR toner. On the other hand, the Al signal is attributed to alumina particles that come from the donor, which is a commercial thermally conductive polyimide that contains alumina filler. The colocation of the oxygen single confirms this interpretation. These inclusions indicate partial transfer of toner- and donor-derived particulates. While they may contribute locally, the fluorescence assays below primarily map antibacterial response versus coating thickness/topography; isolating the specific role of metallic inclusions will be the subject of future work.

To probe how local LINCSAT coating thickness affects bacterial growth, we inoculated *Escherichia coli* (*E. coli*; 4 × 10^7^ CFU mL^–1^) on coated PP blocks for 24 h and quantified viability using a standard live/dead BacLight fluorescence assay. The mean coating thickness for the three levels (Figure 2b) were 38.09 ± 0.15 μm at Level 1 (crest), 16 ± 0.08 μm at Level 2 (shoulder), and 6.78 ± 0.18 μm at Level 3 (outer region). Regions of interest (ROIs) were selected on uncoated PP and within each level (Figure 2c), and the live/dead ratios were calculated. Despite the monotonic decrease in coating thickness from level 1 to level 3, the live/dead ratios exhibit a pronounced nonmonotonic trend (Figure 2d). Uncoated PP shows a high live/dead ratio (20.17 ± 14.16), consistent with minimal bactericidal activity and substantial survival of *E. coli* on hydrophobic polypropylene surfaces reported in prior studies.^24,25^ In contrast, all LINCSAT-coated regions substantially reduced the live/dead ratio (Figure 2d). The intermediate-thickness Level 2 yielded the lowest live/dead ratio (0.883 ± 0.111), compared to level 1 (1.330 ± 0.248) and Level 3 (1.244 ± 0.307). In the statistical comparisons in Figure 2d, Level 2 is significantly lower than Level 1 (*p* < 0.01).

**Figure 2:**
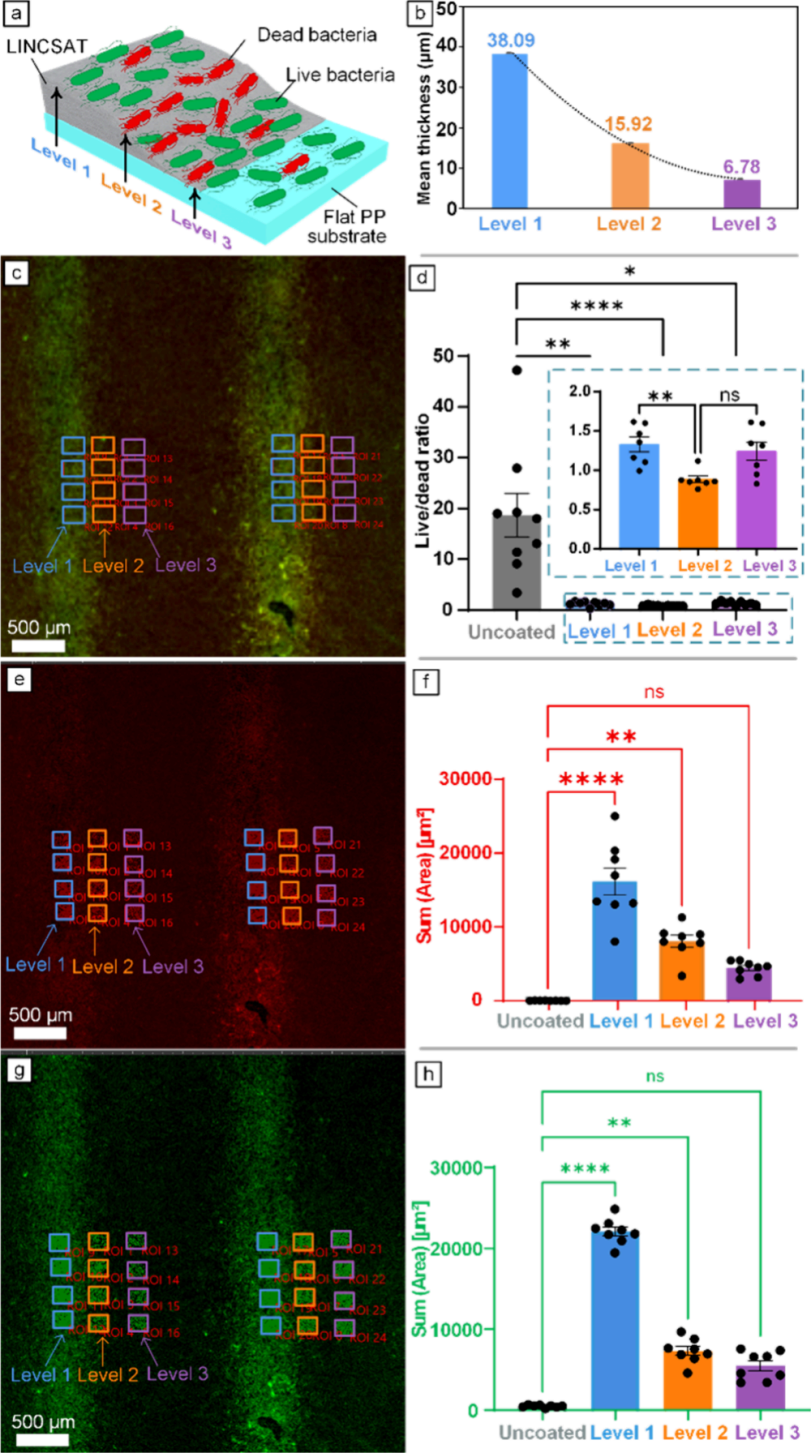
Nonmonotonic dependence of *E. coli* viability and surface-associated coverage on local LINCSAT coating thickness along PP lines on a flat PP surface. (a) Conceptual schematic of antibacterial behavior. (b) Mean coating thickness extracted from 3D profilometry for each level, showing a systematic decrease in average thickness from Level 1 to Level 3. (c) Representative merged live/dead fluorescence image of *E. coli* after incubation on a LINCSAT line, where green and red channels correspond to SYTO 9 stained viable cells and propidium iodide-stained dead cells, respectively. Colored boxes indicate regions of interest (ROIs) assigned to Levels 1–3. (d) Live/dead ratio quantified per ROI for uncoated PP and for each coating-thickness level, showing reduced ratios on coated regions with a minimum at the intermediate Level 2. (e, f) Red-channel image and corresponding summed projected area of dead cells per ROI. (g, h) Green-channel image and corresponding summed projected area of live cells per ROI. Bars show mean ± standard deviation with individual ROI points; statistical significance was evaluated with ns denoting no significant difference and *, **, ***, and **** indicating *p* < 0.05, *p* < 0.01, *p* < 0.001, and *p* < 0.0001, respectively.

To further interpret whether the live/dead ratio changes reflect differences in surface coverage versus viability among surface-associated cells, we quantified the projected fluorescence area per ROI and decomposed it into dead (red) and live (green) components. Total bacterial area was calculated as the sum of the segmented live and dead fluorescence areas (Figure 2e-h). Because these values were obtained from bird’s-eye fluorescence images, a slope correction based on the profilometry-derived coating thickness profile was performed to assess whether projected-area effects influenced the comparison among coating levels (Table S1). Across LINCSAT-coated regions, the total surface-associated bacterial area increased with coating thickness, with the largest total area at level 1 (∼3.8 × 10^4^ μm^2^ per ROI), followed by level 2 (∼1.5 × 10^4^ μm^2^) and level 3 (∼1.0 × 10^4^ μm^2^). The slope correction was negligible, with a maximum surface-area correction factor of 1.0012, corresponding to a 0.12% correction. Applying this correction changed the projected biomass area values by less than 0.13%, so the relative trend among Levels 1–3 remained unchanged (Table S1). Decomposing this total area into dead and live components shows that the fraction of dead biomass is highest at Level 2 (∼52% of the total area), compared with ∼41% and ∼45% at Levels 1 and 3, respectively. Together with the live/dead ratio analysis, these data indicate that the intermediate-thickness coating promotes a stronger reduction in viability among surface-associated bacteria under the same assay conditions, not by reduced overall attachment/coverage. This nonmonotonic response suggests that coating thickness affects both bacteria-surface damage and bacterial retention. At the lowest coating thickness, the LINCSAT layer has fewer accessible porous and asperity-rich nanocarbon features for bacteria-surface interactions, which limit contact-mediated bacterial stress. At the intermediate Level 2 thickness, the coating provides sufficient exposed nanocarbon morphology to promote bacteria-surface interactions, giving the lowest live/dead ratio. Further increasing the coating thickness does not necessarily improve performance because the thicker porous coating can increase the available attachment area and physically retain more bacteria, consistent with prior reports showing that bacterial adhesion and viability on graphitic or rough surfaces depend strongly on surface texture, contact geometry, and accessible area.^14,24,26^ This interpretation is supported by the fluorescence analysis: Level 1 shows the largest total projected bacterial area, whereas Level 2 shows the lowest live/dead ratio and the highest dead-cell fraction.

We next evaluated whether toner-assisted LINCSAT on flat PP blocks can be translated to commercially available PP surgical meshes, whose multifilament architecture, knots, and/or junctions are challenging to coat uniformly.^5,27^ Prior to LINCSAT coating, all meshes (including uncoated controls) were RF plasma cleaned (high-frequency mode, 60 s) to increase surface energy and promote interfacial retention of the transferred coating.^6,8^ All comparisons reported here use identically plasma-cleaned uncoated meshes as controls, ensuring that observed antibacterial differences arise from LINCSAT coatings rather than plasma treatment. For mesh LINCSAT coating (Figure 3a), a toner-coated PI donor was positioned above the mesh with a donor-receiver gap of 1 mm, and the NIR laser was scanned over the donor, at 3.05 W laser power, 200 ns pulse duration, 400 kHz repetition rate, and 80 mm s^–1^ scan speed. To achieve conformal LINCSAT coatings on all sides, each mesh was processed on one side, flipped, and processed again under identical conditions. Optical images show consistent darkening across the mesh (Figure 3b, c), indicating conformal deposition while preserving the mesh architecture. SEM further confirms conformal coverage across the 3D mesh, with individual filaments and knot regions wrapped by a continuous spiky nanocarbon layer without any damage or fiber fusion (Figure 3d-g). SEMs of multiple locations and magnifications (Figure S7) show that this conformal morphology extends to interior junctions and knots. To evaluate coating retention under postprocessing, LINCSAT coated meshes were vortex washed and reimaged by SEM (Figure S8). After washing, the porous nanocarbon morphology remained evident on coated filaments, with no obvious large-area delamination or catastrophic cracking in the surveyed regions. Key spiky features observed prior to washing (Figure 3g) were largely preserved, consistent with retention of the transferred carbon network on PP. Plasma pretreatment also improved postwash coating retention with retained coating mass corresponding to 1.245 ± 0.352% weight increase for untreated PP and 1.635 ± 0.184% for plasma-treated PP after washing (Figure S9). This increase is consistent with the role of increased surface energy in improving interfacial retention.^6,8^ Postwash SEM images further show that the retained coating preserves the porous, spiky LINCSAT morphology on both mesh and flat PP substrates in the surveyed regions (Figures S6 and S8). To further assess the LINCSAT-PP interface on planar substrates, a modified tape-peel test based on ASTM D3359 was performed on flat PP blocks before and after washing (Figure S15). Optical images showed partial transfer of black nanocarbon to the adhesive tape, indicating removal of loosely attached material, while SEM images confirmed that porous LINCSAT morphology remained on both unwashed and washed flat PP surfaces after peeling. These observations indicate that LINCSAT can produce wash-resilient porous nanocarbon coverage on both mesh and flat PP substrates without added binders, although loosely attached material can be removed under adhesive peeling. While coating coverage was confirmed in Figures 3, S7, and S8, local variations were still observed because of the curved mesh filaments, knots, and raster-line overlap. In contrast to flat PP blocks, where the continuous ridge profile enabled profilometry-defined Level 1–3 analysis, the mesh geometry required classification by optical contrast. Accordingly, mesh regions were grouped as relatively thick or thin coating areas (Figure 4a, b) and compared with uncoated PP mesh. To further evaluate coating formation as a function of local surface orientation, LINCSAT was applied to individual PP suture fibers with diameters of 150 and 500 μm under the same mesh-coating conditions (Figure S10). SEM images acquired from defined circumferential positions show porous LINCSAT-derived nanocarbon at the donor-facing apex, lateral sides, and lower opposite region of both fibers. The coating appears thicker near more exposed regions and thinner in less directly exposed regions but remains evident around the circumference. These observations support coating transfer to curved PP surfaces with different local orientations and are consistent with deposition from a laterally spreading transfer plume.

**Figure 3:**
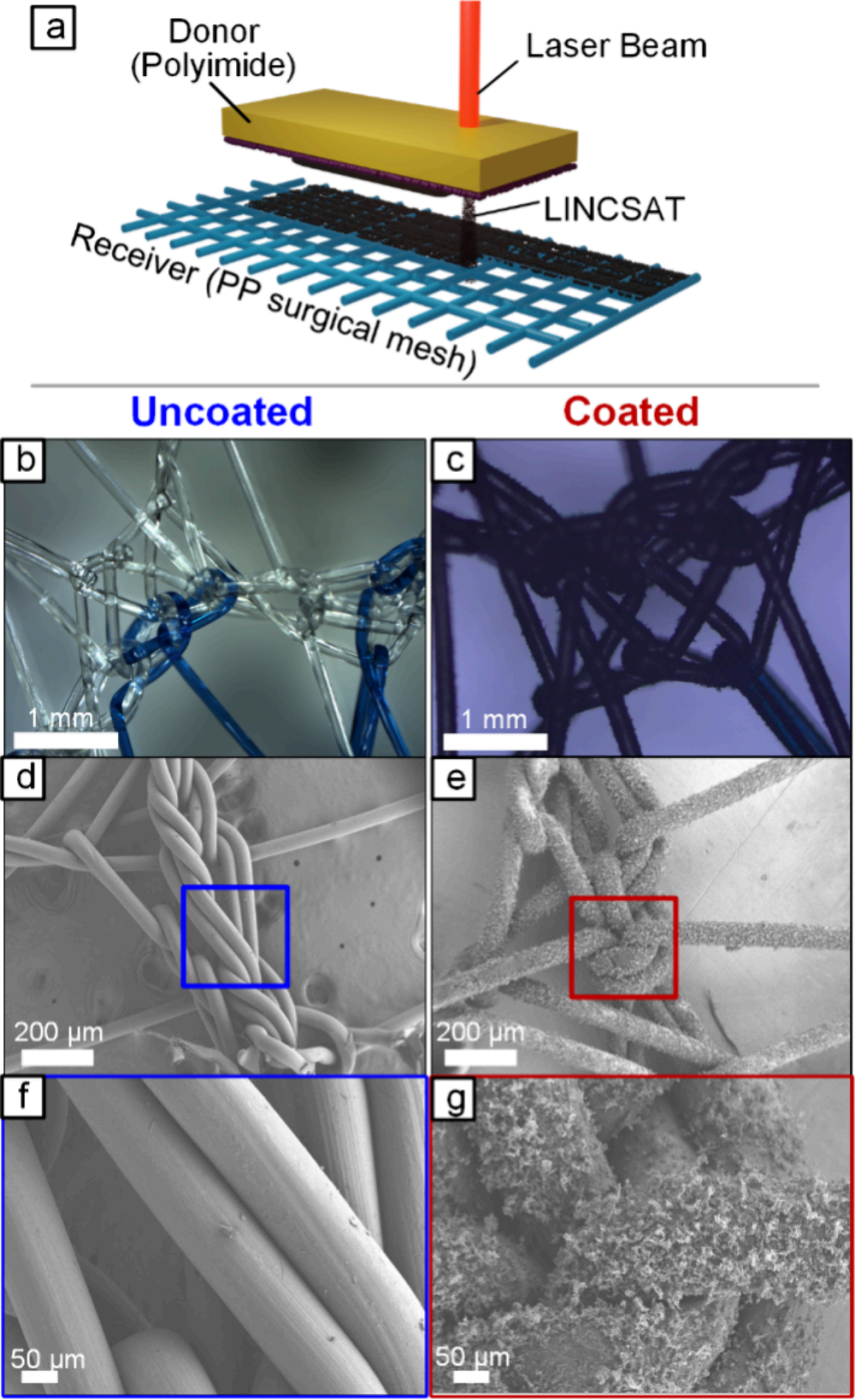
Conformal LINCSAT coating on a 3D polypropylene surgical mesh. (a) Schematic of the toner-assisted LINCSAT configuration used to coat commercial PP surgical mesh. (b, c) Optical micrographs of the mesh before and after coating, showing uniform coating of the fiber and junctions without melting or distortion. (d, e) Low-magnification SEM images of uncoated and coated meshes, respectively, illustrating that the LINCSAT process deposits a continuous coating along individual fibers and around junctions without fusing or collapsing the mesh. Green boxes indicate regions magnified in panels (f, g). (f) High-magnification SEM of uncoated PP fibers, showing smooth, featureless surfaces. (g) High-magnification SEM of coated fibers, revealing a conformal, porous graphene-like nanocarbon layer wrapped around each filament/knot.

**Figure 4:**
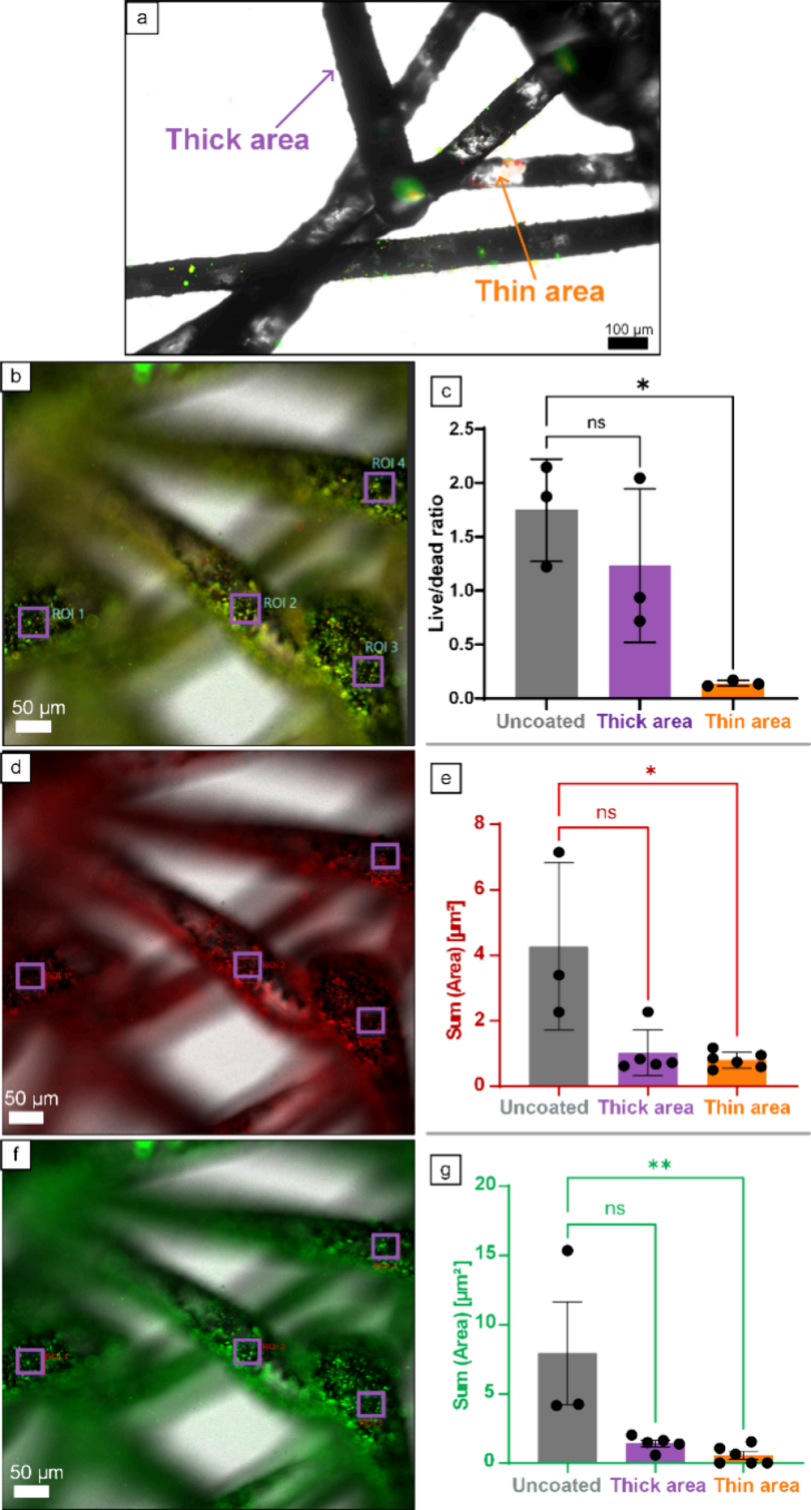
Thickness-dependent *E. coli* response on LINCSAT-coated PP surgical mesh. (a) Live/dead fluorescence overlay of a LINCSAT-coated mesh region, showing qualitative thick and thin areas of the graphene-like coating along different filaments. (b) Higher magnification merged live/dead image with representative regions of interest (ROIs). (c) Live/dead ratio extracted from the fluorescence images for each condition (uncoated control, thick-coated, thin-coated), showing the lowest live/dead ratio on thin-coated regions compared to thick-coated and uncoated controls. (d, e) Red-channel image and summed projected areas of dead cells per ROI. (f, g) Green-channel image and summed projected areas of live cells per ROI. Bars: mean ± standard deviation.

Polypropylene meshes were inoculated with *E. coli* (4 × 10^7^ CFU mL^–1^) and incubated for 24 h, after which bacterial viability was quantified using a live/dead BacLight fluorescence assay. Thin-coated regions exhibited the lowest live/dead ratio (0.141 ± 0.02), significantly lower than uncoated mesh (1.75 ± 0.47, *p* < 0.05; Figure 4c). Thick regions showed an intermediate and more variable response (1.23 ± 0.71) and were not significantly different from uncoated (ns). The larger ROI-to-ROI scatter, particularly for thick regions, suggests spatial variability in large porous structures that affects bacteria attachment and viability. These results demonstrate that coating-thickness control is a key parameter for improving LINCSAT antibacterial performance on complex 3D mesh substrates.

Quantitative image analysis of surface-associated *E. coli* on the mesh filaments (Figure 4d-g) shows that the total segmented fluorescence area per area ROI (dead + live) is highest on uncoated PP mesh (12.2 ± 8.9 μm^2^ per ROI) and is reduced on LINCSAT coated regions. Thick and thin coated segments exhibited lower total coverage (2.44 ± 1.01 and 1.38 ± 0.79 μm^2^ per ROI, respectively), corresponding to ∼5× and ∼9× reductions relative to uncoated mesh. Decomposing the total area into channel-specific contributions indicates that both dead and live projected areas decrease on coated regions (Figure 4e, g). Live coverage is lowest on thin regions (0.59 ± 0.60 μm^2^ per ROI) compared with thick regions (1.42 ± 0.53 μm^2^ per ROI; *p* < 0.01) and uncoated mesh (7.91 ± 6.44 μm^2^ per ROI), consistent with reduced viable biomass on the thinnest coatings. Together, the combined analysis support that thin LINCSAT coated mesh regions most effectively suppress surface-associated colonization and viable biomass under these assay conditions.

To qualitatively assess how LINCSAT coatings influence bacterial colonization on the 3D mesh, we performed agar-plate attachment assays. Uncoated and LINCSAT coated PP meshes were placed on nutrient agar lawns of *E. coli* (Figure 5) and *S. aureus* (Figure S11) prepared by spreading a suspension at 4 × 10^7^ CFU mL^–1^ and incubating for 24 h. After incubation, bacterial growth was visible around and within the mesh footprints for both uncoated and coated samples (Figure 5a-d), indicating that bacteria readily access the mesh architecture under these conditions. In contrast, the coated mesh exhibited a qualitatively reduced growth/coverage halo for *E. coli* relative to uncoated controls (Figure 5d). In contrast, the *S. aureus* plates (Figure S11) did not show an analogous reduction around the coated mesh. Because this assay is qualitative and integrates effects of attachment, growth, and local nutrient transport, we use it primarily as a visual screening test rather than quantitative measure of adhesion. The fluorescence-based viability/coverage assays (Figures 2 and 4) therefore provide the primary basis for comparing antibacterial performance of LINCSAT.

**Figure 5:**
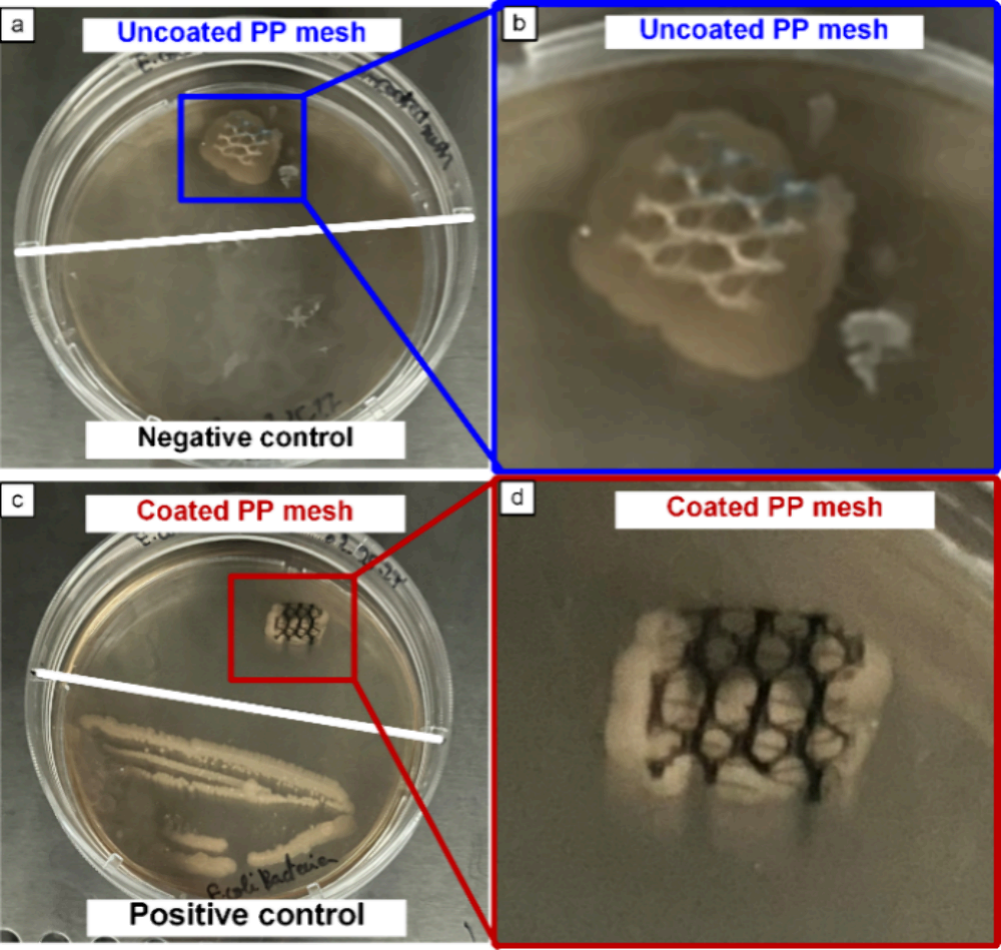
Bacterial attachment on PP mesh with and without LINCSAT coating. (a, b) Agar plate seeded with an *E. coli* lawn (4 × 10^7^ CFU mL^–1^, 24 h incubation) containing an uncoated PP mesh, used as the negative control (mesh without antibacterial LINCSAT coating); (b) magnified view of the mesh region. (c, d) Agar plate prepared under identical conditions but containing a LINCSAT-coated PP mesh, used as the positive control for an antibacterial surface treatment; (d) zoomed-in view showing the dark, conformal carbon network along the mesh struts. The streaked region at the bottom of each plate corresponds to the *E. coli* inoculum and serves only as general growth control.

To characterize how LINCSAT modifies PP wettability, we measured static and dynamic water contact angles on flat PP blocks and static water contact angles on PP mesh before and after washing (Figures S12–S14). On flat PP blocks processed under the same coating conditions (Figure S12) uncoated PP exhibited a static contact angle of 105.84 ± 0.58° (Figure S12 a), consistent with the hydrophobic nature of polypropylene surfaces reported in the literature.^28^ After LINCSAT coating, the apparent static contact angle increased to 156.13 ± 0.31°, indicating a superhydrophobic state. Following two vortex washing cycles, the static contact angle decreased slightly to 137.98 ± 0.65°, but remained substantially higher than uncoated PP, consistent with retention of a rough, hydrophobic coating layer after washing. Dynamic contact-angle measurements (Figure S12 b) further differentiated these wetting states, uncoated PP showed advancing/receding angles of 110.66 ± 4.17° and 95.38 ± 2.83° (hysteresis 15.3 ± 5.47°), whereas unwashed LINCSAT coated PP exhibited 156.96 ± 1.64° and 156.96 ± 1.64.° (hysteresis 0°), consistent with a low-hysteresis superhydrophobic regime often associated with Cassie–Baxter-like wetting on hierarchical roughness.^29^ After washing, the advancing angle remained high (144.7 ± 0.28°), whereas the receding angle decreased (103.87 ± 0.25°), resulting in a much larger hysteresis of 40.8 ± 0.52°. This increase in hysteresis, despite the still-elevated static contact angle, suggests stronger droplet pinning after washing while maintaining overall high apparent wettability (i.e., enable switching from superhydrophobic to parahydrophobic surfaces).^30^ To evaluate whether the wetting response continued to decrease with further washing, LINCSAT-coated PP blocks were subjected to five consecutive vortex-washing cycles under the same conditions. The largest change occurred after the first cycle, where the static contact angle decreased from 156.13 ± 0.31° to 139.02 ± 0.44°. Additional washing produced only minor changes, with contact angles of 137.82 ± 0.26°, 137.79 ± 0.89°, 136.18 ± 0.69°, and 136.77 ± 0.68° after two, three, four, and five cycles, respectively (Figure S13). These results indicate that the first wash produces the dominant change in the wetting state, likely by removing the loosely attached surface material, after which the coating retains stable hydrophobic behavior through five washing cycles. Because PP mesh is a curved and open architecture, contact angles measured on mesh are interpreted as apparent geometry-dependent values rather than intrinsic values directly interchangeable with flat PP measurements. Nevertheless, the same overall trend was observed on mesh: the apparent contact angle increased from 119.59 ± 0.19° for uncoated PP mesh to 146.27 ± 1.15° after LINCSAT coating and remained highly hydrophobic after washing at 136.32 ± 0.51° (Figure S14). Thus, curvature changes the absolute apparent contact-angle value, but the coating-induced increase in hydrophobicity is preserved on the 3D mesh. Overall, these wettability results provide mechanistic context for the antibacterial assays by describing how liquid can access the porous LINCSAT coating. Before washing, the high apparent contact angle and negligible hysteresis indicate limited liquid adhesion to the rough nanocarbon network. After washing, the coating remains hydrophobic, but the increased hysteresis indicates stronger pinning and greater local liquid interaction with the porous structure. This distinction is important because bacteria interact with the wetted and accessible fraction of the coating rather than with the apparent macroscopic contact angle alone. Therefore, contact angle is not treated as a direct predictor of antibacterial performance; instead, it describes the liquid-access environment in which bacterial attachment and viability occur. This interpretation is consistent with prior reports showing that bacterial adhesion depends on wetting state, surface texture, contact geometry, and accessible surface area.^24,25,29^ The fluorescence-based live/dead and projected-area analyses therefore provide the direct evidence for antibacterial performance, while the wettability measurements help explain how the LINCSAT coating may regulate bacteria-surface contact.

In conclusion, toner-assisted LINCSAT provides a one-step, versatile route to deposit spiky nanocarbon coatings onto PP without thermal damage. By decoupling the carbon precursor (donor) from the target surface (receiver), LINCSAT enables nanocarbon coating on nongraphitizing, temperature-sensitive polyolefins that are not compatible with direct LIG writing. On flat PP blocks, LINCSAT produces continuous porous ridges (t_avg_ ≈ 30–38 μm) with Raman signatures consistent with disordered sp^2^-enriched carbon and a reproducible lateral thickness gradient that can be discretized into crest, shoulder, and flank levels. The coating also produces a pronounced shift in wettability to superhydrophobic regime. Spatial mapping of *E. coli* response across the thickness levels reveals a nonmonotonic dependence on coating thickness, with an intermediate thickness minimizing the live/dead ratio and reducing surface-associated viable biomass under the tested assay conditions. On commercial PP surgical mesh, LINCSAT yields conformal porous coverage of filaments and knot regions while preserving the macroscopic open mesh architecture, and the spiky coating morphology is retained after vortex washing. Fluorescence-based assays further indicate reduced viability and reduced surface-associated coverage in thin coated regions relative to uncoated mesh. Collectively, these results establish LINCSAT as a platform for conformal nanocarbon coatings on low-temperature thermoplastics and identify coating thickness as an explicit design parameter for antibacterial performance on complex 3D substrates.

## Supplementary Material



## Notes

Slope correction analysis for projected biomass-area quantification on LINCSAT-coated PP blocks (Table S1); additional optical images of LINCSAT line patterns on PP blocks at different laser powers (Figure S1); Raman spectra of LINCSAT-written nanocarbon lines at different powers, including PP C–H stretching contributions in the 2800–3000 cm^–1^ region (Figure S2); SEM-EDS elemental maps of LINCSAT lines highlighting carbon uniformity and localized oxygen/iron/aluminum associated with transferred inclusions (Figure S3); Raman spectra comparing LINCSAT-coated PP across three coating-thickness levels (Figure S4); SEM images comparing LINCSAT-coated PP block morphology across three coating-thickness levels (Figure S5); SEM images of LINCSAT-coated flat PP blocks after five washing cycles (Figure S6); extended SEM survey demonstrating LINCSAT coating coverage on PP mesh across multiple knots and fiber junctions (Figure S7); SEM characterization of coated PP mesh after washing showing coating retention (Figure S8); effect of oxygen plasma cleaning on LINCSAT coating mass retention quantified by postwash percent weight increase (Figure S9); orientation-resolved SEM analysis of LINCSAT-coated PP fibers with diameters of 150 and 500 μm to evaluate coating coverage around curved surfaces with different local orientations (Figure S10); *S. aureus* attachment assays on uncoated and LINCSAT-coated meshes (Figure S11); static and dynamic water contact-angle measurements on uncoated and LINCSAT-coated PP blocks before and after washing (Figure S12); effect of repeated washing cycles on the wettability of LINCSAT-coated PP (Figure S13); static water contact-angle measurements on uncoated and LINCSAT-coated PP mesh before and after washing (Figure S14); and modified tape-peel adhesion testing of LINCSAT-coated PP before and after washing (Figure S15) (PDF)

## ABBREVIATIONS

LINCSAT: laser-induced nanocarbon synthesis and transfer
LIG: laser-induced graphene
PP: polypropylene
PP mesh (Gynemesh): polypropylene surgical mesh
PI: polyimide
MOPA: master-oscillator power-amplifier (fiber laser)
NIR: near-infrared
MICR ink: magnetic-ink character recognition (iron-oxide-bearing) ink
SEM: scanning electron microscopy
Raman (D, G, 2D): Raman bands
CA: contact angle
Adv/Rec: advancing/receding (contact angle)
CAH: contact-angle hysteresis
ROI: region of interest
H­(avg): average thickness
3D: three-dimensional
*E. coli*: *Escherichia coli*
