# Supplementary material for: Antimicrobial Coating of Surgical Meshes by Laser-Induced Nanocarbon Synthesis and Transfer
Source: ACS Appl Polym Mater. 2026 Jun 8;8(12):9028–36. doi: 10.1021/acsapm.6c00879 (PMC13316859; doi:10.1021/acsapm.6c00879)
Supplement: Supplementary file 1 [file ap6c00879_si_001.pdf]

Supporting Information (SI)

for

Antimicrobial Coating of Surgical Meshes by Laser-Induced Nanocarbon Synthesis and Transfer

Diala Bani Mustafa[1, ‡], Josiane Semaan[2, ‡], Soumalya Ghosh[1], Nathan Carney[1], Tagbo H.R. Niepa[3], Rui Liang[2*], and Mostafa Bedewy[1,4,5*].

[1] Department of Mechanical Engineering and Materials Science, University of Pittsburgh, PA 15261, USA

[2] Department of Anesthesiology, The Ohio State University, Columbus, OH 43210, USA

[3] Department of Chemical Engineering, Department of Biomedical Engineering, Carnegie Mellon University, Pittsburgh, PA, 15213, USA

[4] Department of Chemical and Petroleum Engineering, University of Pittsburgh, Pittsburgh, PA 15261, USA

[5] Department of Industrial Engineering, University of Pittsburgh, Pittsburgh, PA 15261, USA

‡ Equal contribution

Corresponding Author

*Mostafa Bedewy (mbedewy@pitt.edu)
*Rui Liang (liang.1543@osu.edu)

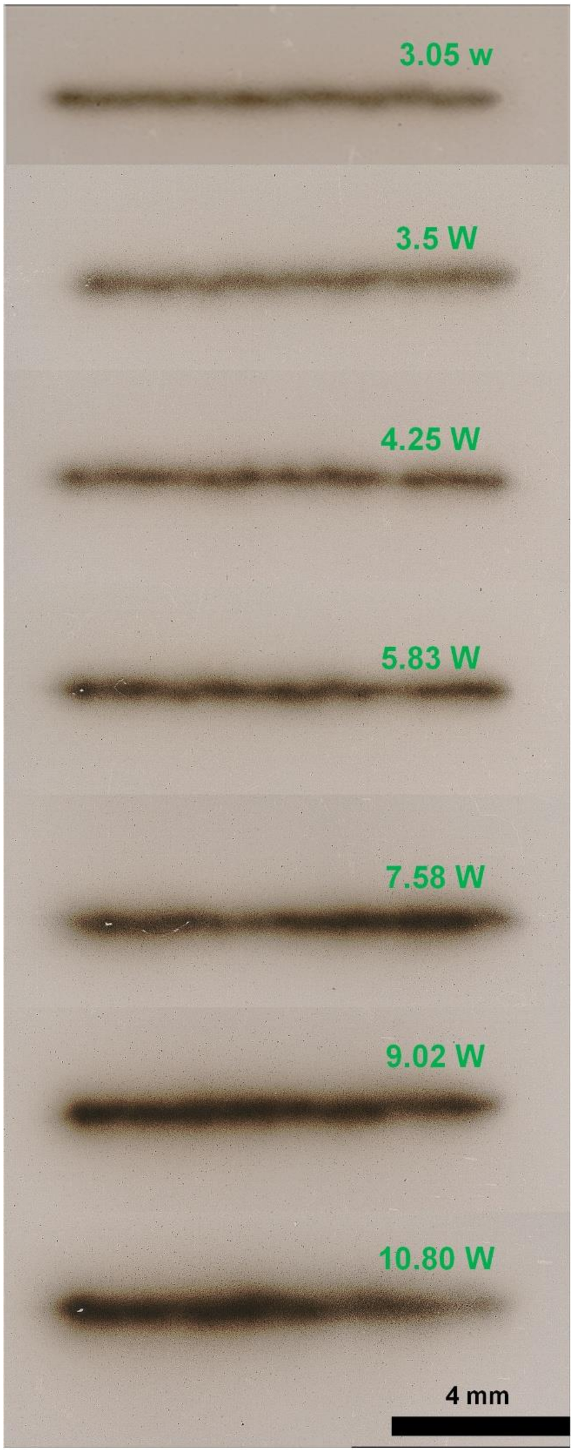

Figure S1. Optical image of nanocarbon lines fabricated by LINCSAT from the toner-coated polyimide donor to the PP block receiver at various powers.

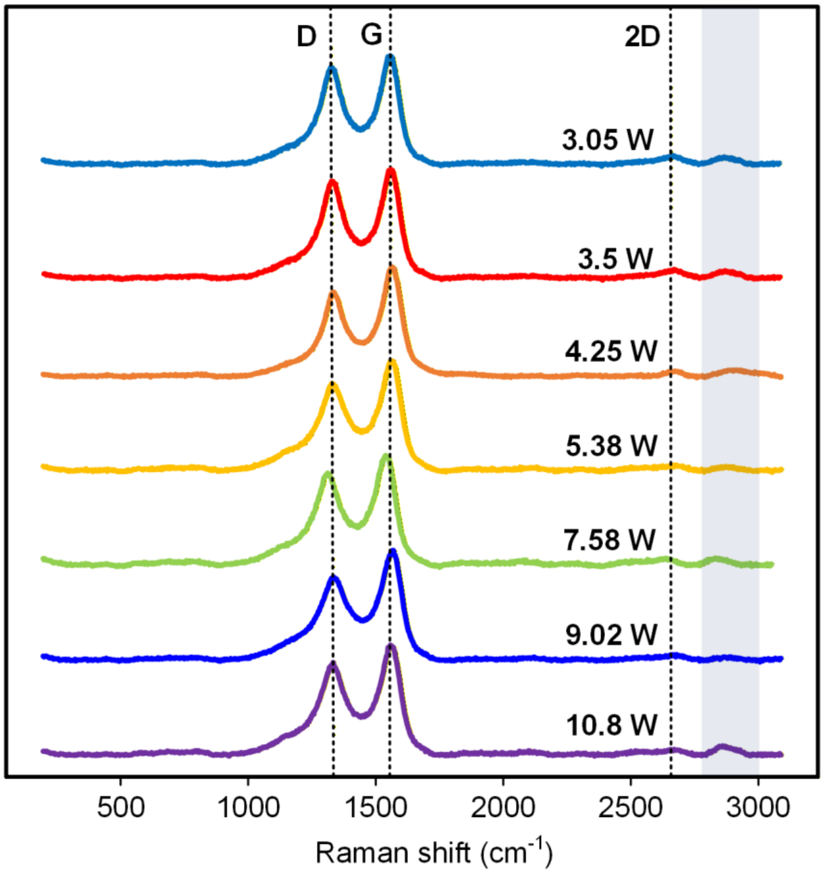

Figure S2. Raman spectra acquired from LINCSAT-written nanocarbon line patterns produced at increasing laser power (3.05-10.8 W). The characteristic D (~1350 cm^{-1}) and G (~1580 cm^{-1}) bands are observed for all conditions, with a weak 2D feature near ~2700 cm^{-1} (dashed reference). The shaded region (2800–3000 cm^{-1}) corresponds to polypropylene C–H stretching (CH_2/CH_3) modes from the underlying PP substrate.

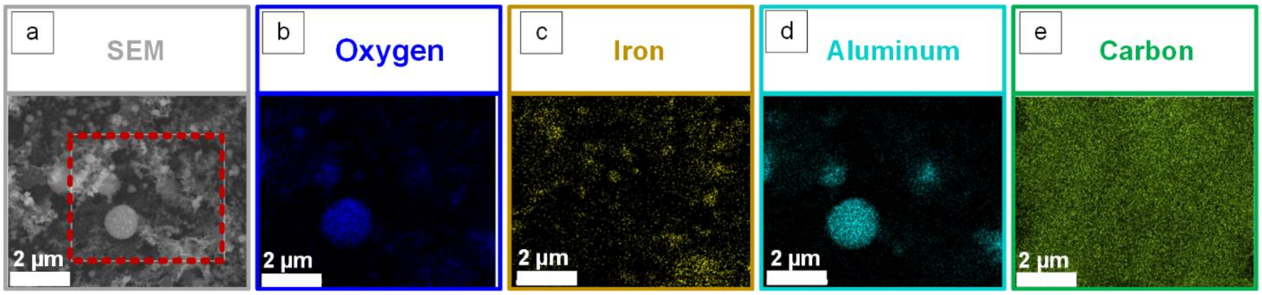

Figure S3. SEM and EDS elemental mapping of the LINCSAT lines on PP block processed at 3.05 W (shown in Figure 1 and Figure S1). (a) SEM image of the analyzed region (red dashed box). Corresponding elemental maps for (b) oxygen, (c) iron, (d) aluminum, and (e) carbon show that the 3.05 W line exhibits a uniform carbon distribution across the surface, while localized enrichment of oxygen, iron, and aluminum indicates compositional heterogeneity associated with LINCSAT process resulting in transferring alumina particles from the commercial thermally conductive polyimide and the iron oxide from the toner.

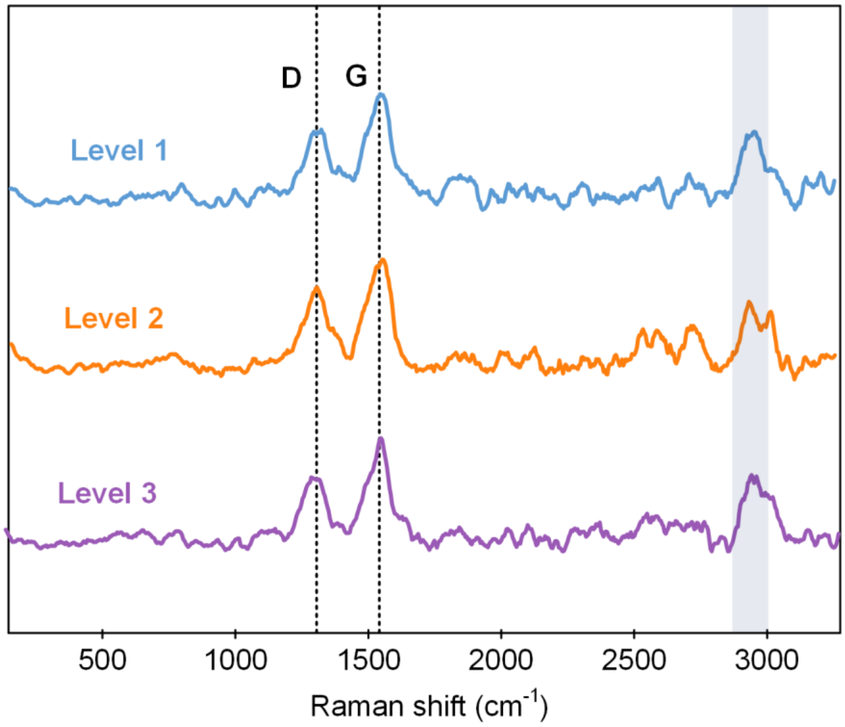

Figure S4. Raman spectra of LINCSAT-coated PP across three coating-thickness levels. Raman spectra acquired from Level 1, Level 2, and Level 3 of a representative LINCSAT line on PP, corresponding to the crest, shoulder, and outer region of the coating, respectively. All three levels show characteristic D (~1350 cm^{-1}) and G (~1580 cm^{-1}) bands of disordered sp^2-rich nanocarbon. The shaded region (2800-3000 cm^{-1}) corresponds to polypropylene C-H stretching modes (CH_2/CH_3) from the underlying PP substrate. No systematic shift in the D or G band position is observed across the three levels, indicating that the lateral thickness gradient is primarily associated with coating buildup/coverage rather than an obvious position-dependent change in carbon structure.

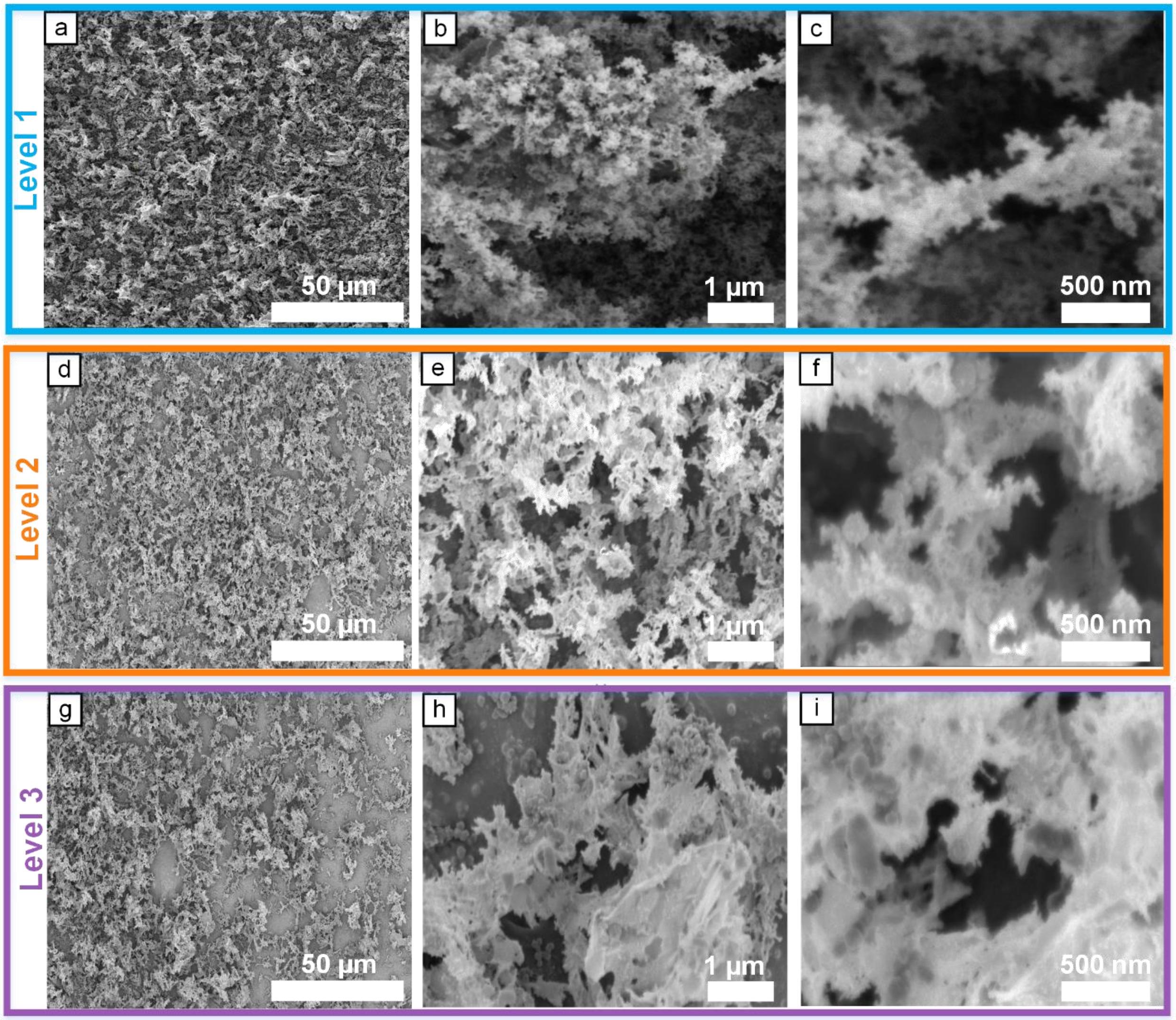

Figure S5. SEM images of LINCSAT-coated PP block at different coating-thickness levels. SEM images acquired from Level 1 (a-c), Level 2 (d-f), and Level 3 (g-i), corresponding to the crest, shoulder, and outer region of a representative LINCSAT line, respectively. The coating shows a porous nanocarbon morphology across all three levels, with interconnected nanoscale aggregates and open voids. No obvious change in the characteristic nanoscale morphology is observed among the three levels, while the apparent coating thickness varies laterally with position along the line.

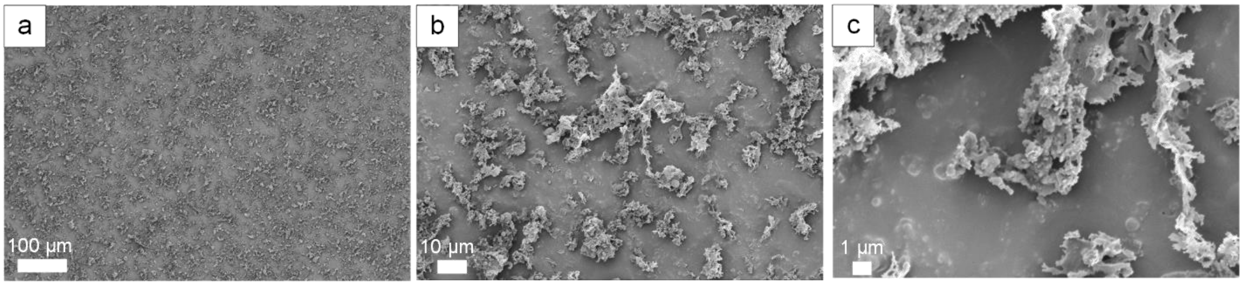

Figure S6. SEM images of LINCSAT-coated flat PP block after 5 cycles of washing, showing retention of the transferred nanocarbon coating on the planar substrate. (a-c) Multiscale views of the washed PP surface reveal that the laser-induced nanocarbon layer remains present after vortex washing, with porous aggregates, open voids, and spiky nanoscale features still visible on the substrate. These images confirm that the characteristic LINCSAT morphology is retained after washing on flat PP, although local variations in coating coverage are observed.

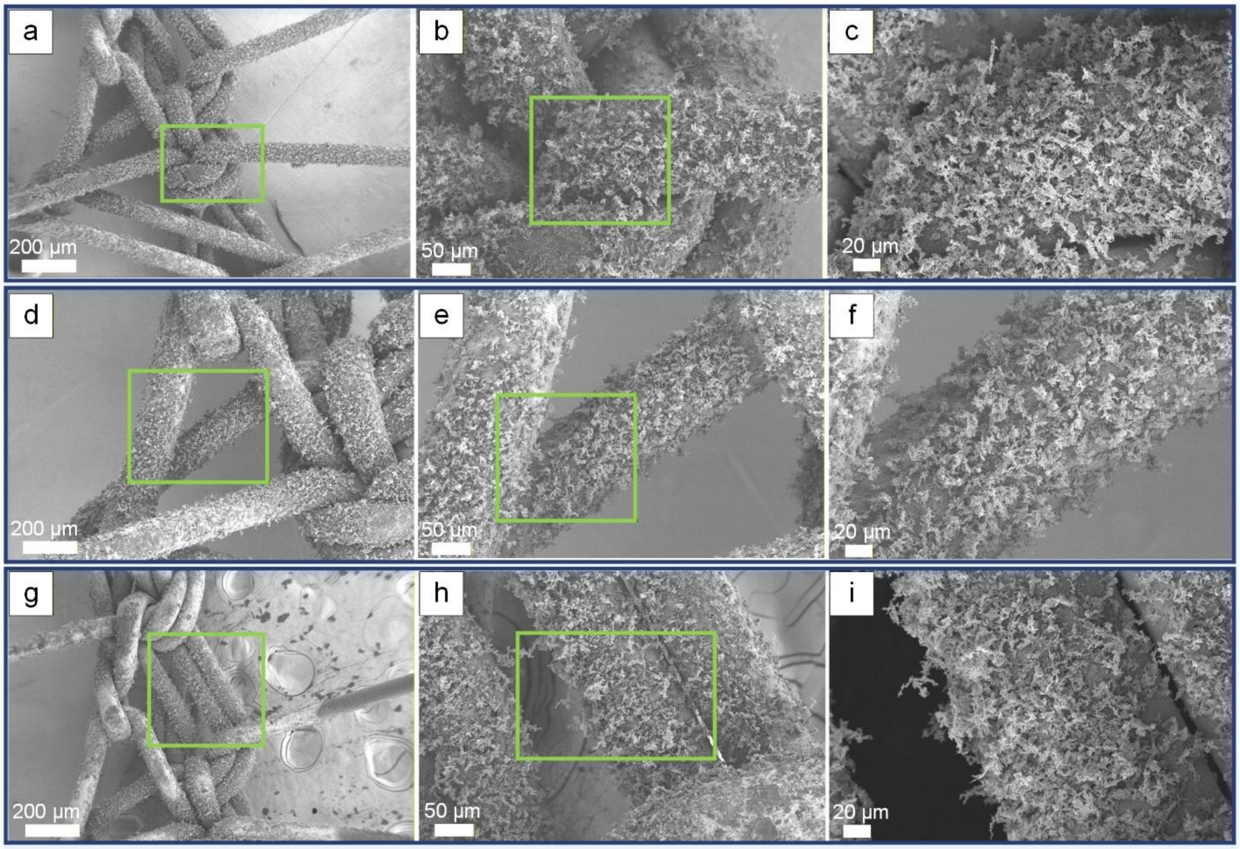

Figure S7. SEM images showing conformal carbon coating on gynecological polypropylene mesh processed by the LINCSAT method. (a–c), (d–f), and (g–i) correspond to different knot and fiber junction locations at increasing magnifications. The images demonstrate uniform and conformal coating of the intertwined fibers, including complex 3D junctions, indicating effective laser-induced nanocarbon synthesis and transfer even at occluded and curved regions of the mesh structure.

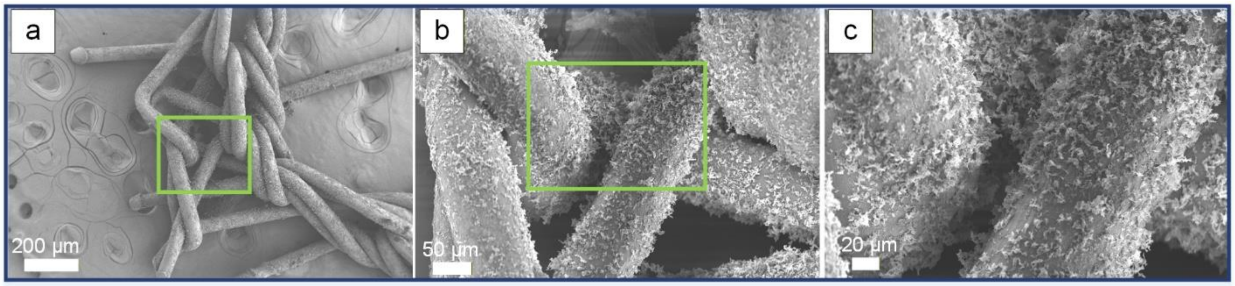

Figure S8. SEM images of polypropylene mesh after washing, showing the robustness of the conformal coating formed by the LINCSAT process. (a-c) Multiscale views of the same mesh region reveal that the laser-induced nanocarbon layer remains uniformly adhered to the fibers and knot junctions even after washing, confirming strong interfacial bonding and coating stability on the 3D fibrous architecture.

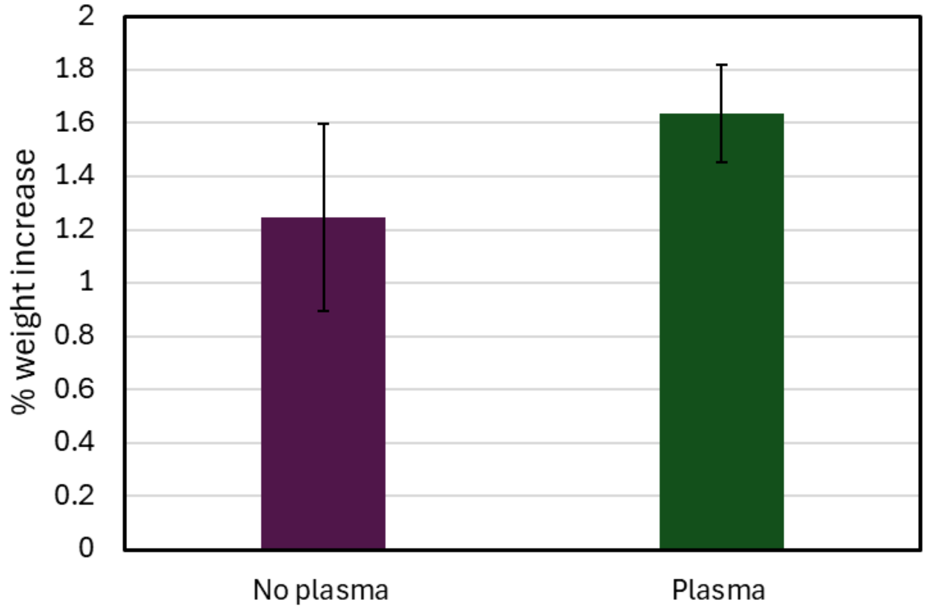

Figure S9. Effect of plasma cleaning on transferred LINCSAT coating mass retention. Average % weight increase after LINCSAT and washing for substrates without plasma treatment (purple) and with 60 s oxygen plasma cleaning (green). These results show the enahanced interfacial bonding of LINCSAT coating with PP mesh surface as a result of the plasma treatement prior to LINCSAT process.

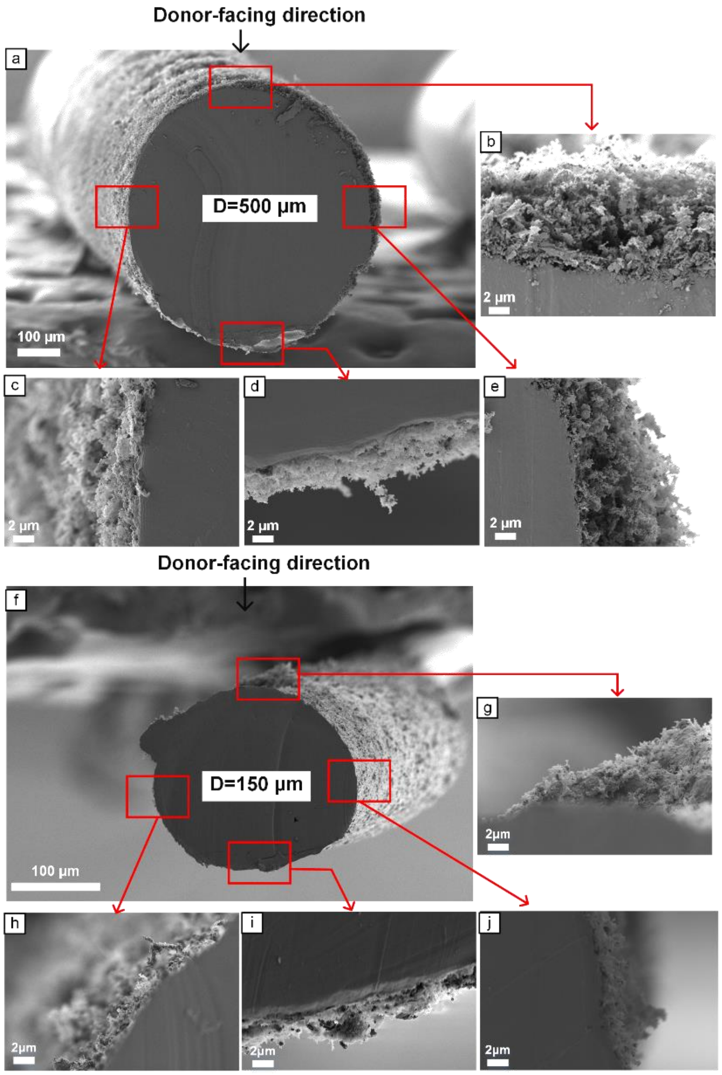

Figure S10. Orientation-resolved SEM analysis of LINCSAT coating on polypropylene suture fibers. (a) Low-magnification SEM image of a LINCSAT-coated PP fiber with a diameter of 500 μm processed at 3.05 W laser power, 200 ns pulse duration, 400 kHz repetition rate, and a 1 mm donor-receiver gap. The arrow indicates the donor-facing direction, and red boxes mark circumferential locations selected for higher-magnification imaging. (b-e) Higher-magnification SEM images acquired from the donor-facing apex, lateral surfaces, and lower/opposite circumferential region of the 500 μm fiber, showing porous nanocarbon coating at multiple local orientations. (f) Low-magnification SEM image of a LINCSAT-coated PP fiber with a diameter of 150 μm processed under the same conditions. (g-j) Higher-magnification SEM images acquired from the donor-facing apex, lateral surfaces, and lower/opposite circumferential region of the 150 μm fiber. The presence of porous nanocarbon at multiple circumferential locations supports coating formation on accessible curved PP surfaces with different local orientations relative to the donor plane.

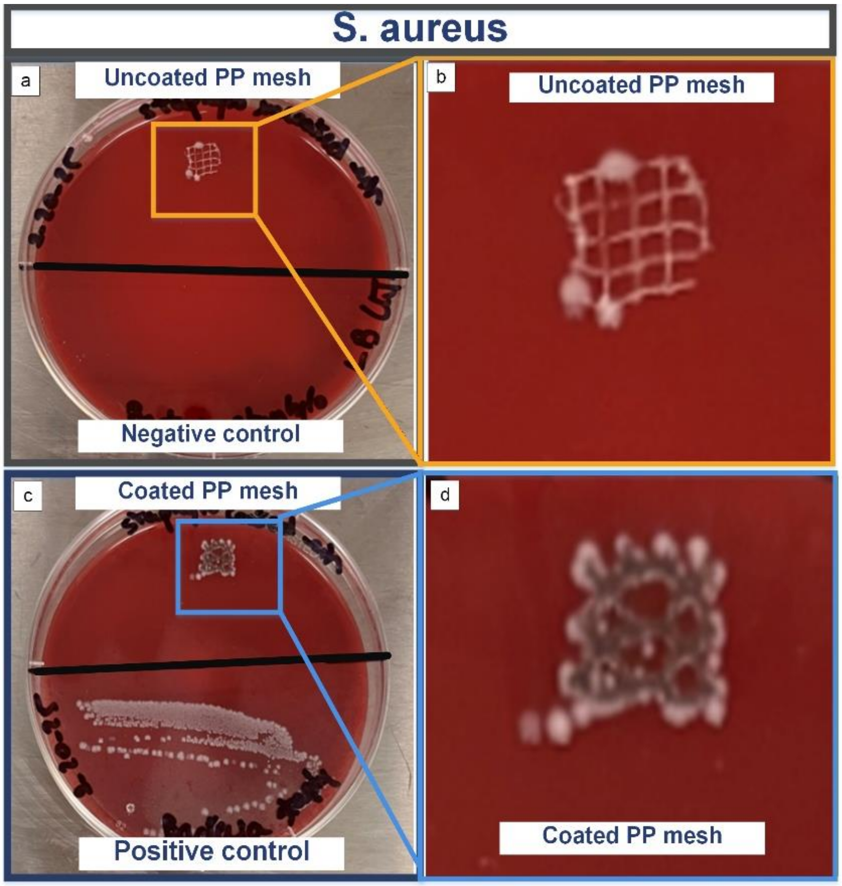

Figure S11. (a-d) Bacterial attachment assay of uncoated and LINCSAT-coated polypropylene meshes against Staphylococcus aureus. Both coated and uncoated meshes show evidence of bacterial attachment and colony growth after spreading a suspension at ≈ 4.105× 10^7 CFU mL^{-1} on nutrient agar and incubating the plates for 24 h.

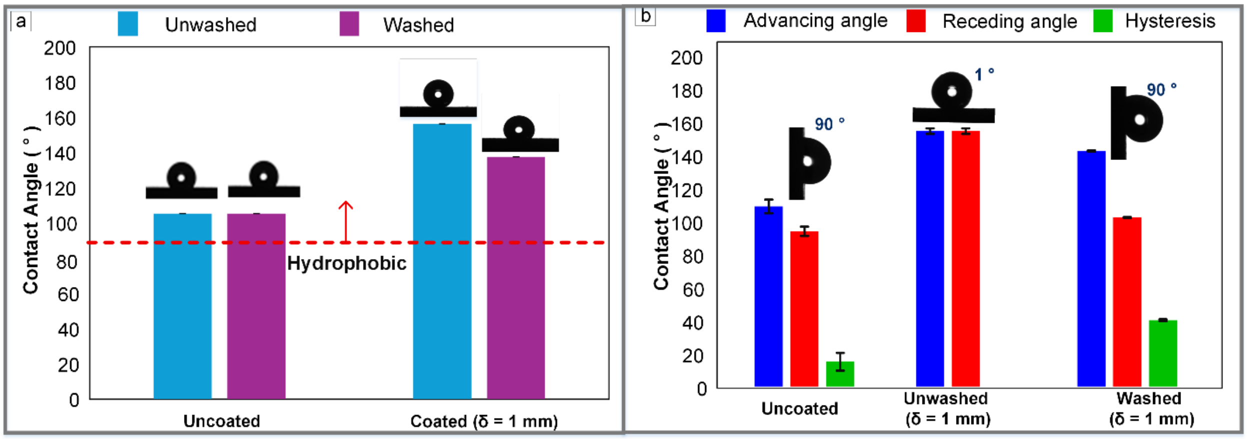

Figure S12. Wettability of LINCSAT-coated PP blocks before and after washing. (a) Static water contact angle measured on uncoated PP and LINCSAT-coated PP blocks (δ = 1 mm) in the unwashed and vortex-washed states. (b) Dynamic contact angle measurements showing advancing and receding angles and the resulting contact-angle hysteresis for uncoated PP and for LINCSAT-coated PP blocks before and after washing. Bars show mean ± standard deviation.

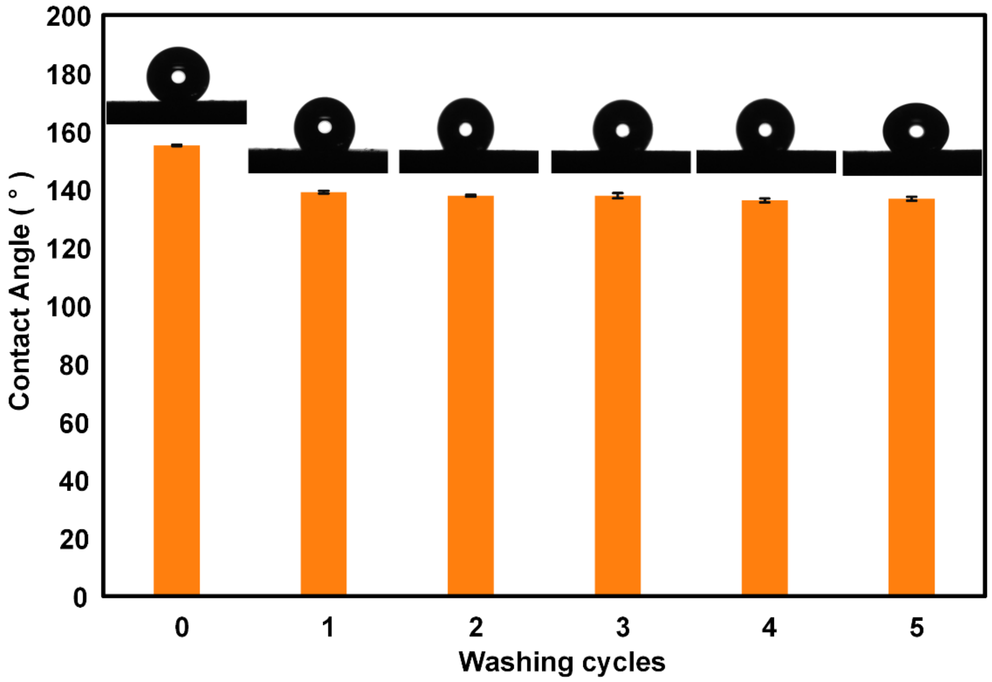

Figure S13. Effect of repeated washing cycles on the wettability of LINCSAT-coated PP. Static water contact angles measured on LINCSAT-coated flat PP blocks after sequential washing cycles. Representative droplet images are shown above each bar. The unwashed coating showed a contact angle of 156.13± 0.31°. After the first washing cycle, the contact angle decreased to 139.02 ± 0.44° and then remained within a narrow range of approximately 136-138° through five washing cycles, indicating that the coated PP surface retained hydrophobic wetting behavior after repeated washing.

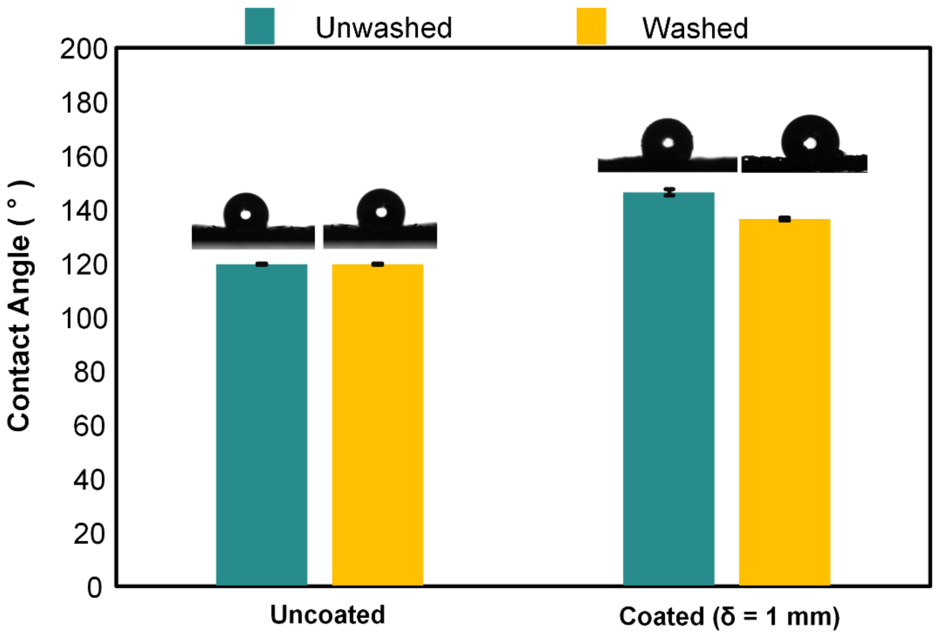

Figure S14. Wettability of uncoated and LINCSAT-coated PP surgical mesh before and after washing. Static water contact angles measured on uncoated PP mesh and LINCSAT-coated PP mesh prepared at δ = 1 mm. Representative droplet images are shown above the corresponding bars. The LINCSAT-coated mesh exhibits a higher apparent contact angle than the uncoated mesh, and the coated mesh remains hydrophobic after washing.

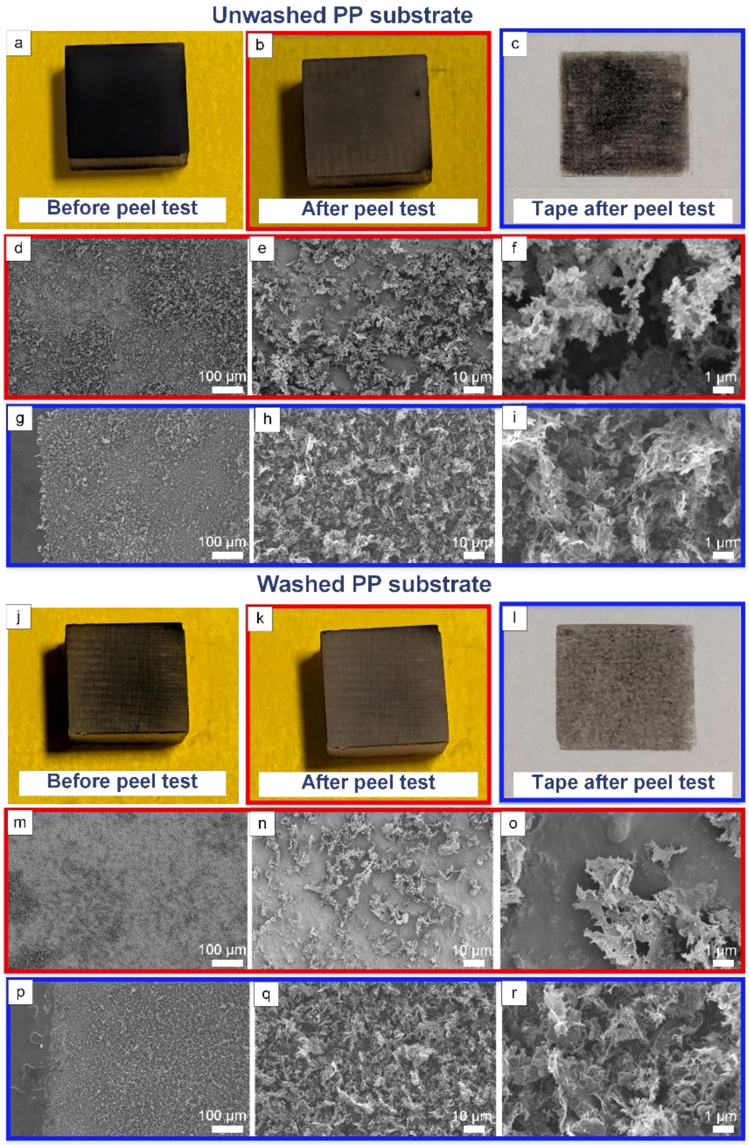

Figure S15. Modified tape-peel adhesion test of LINCSAT-coated PP based on ASTM D3359. Optical images show the coated PP substrate before peel testing, the PP substrate after peel testing, and the tape surface after peeling for the unwashed sample (a-c) and washed sample (j-l). SEM images of the unwashed PP surface after peeling (d-f) and the corresponding tape surface (g-i) show partial removal of carbonaceous material while porous nanocarbon features remain on the PP substrate. SEM images of the washed PP surface after peeling (m-o) and the corresponding tape surface (p-r) similarly show transferred coating fragments on the tape, with retained porous nanocarbon morphology on the PP surface. These results indicate that the tape peel test removes loosely attached material but does not cause complete coating delamination from either unwashed or washed LINCSAT-coated PP substrate.

Table S1. Assessment of slope-related projection effects in biomass-area quantification. Average coating thicknesses and lateral positions measured by profilometry were used to estimate the slope angle between LINCSAT coating regions and the corresponding surface-area correction factor, $S=A_{slope}/A_{proj}$. The maximum correction was 0.12%, and applying the correction to the projected biomass area values produced negligible changes, confirming that the biomass-area trends are not affected by the coating slope.

Level	Coating thickness (µm)	Distance from ridge center (µm)	Thickness difference, Δz (µm)	Lateral distance Δx (µm)	Slope angle (°)	Surface area correction, S	Surface-area correction (%)	Biomass area correction factor	Projected biomass area (bird's-eye fluorescence ROI) (µm²)	Slope-corrected biomass area (µm²)
1	38.09	150	22.17	452	2.808031543	1.001202164	0.120216354	0.99879928	38199.28125	38153.41461
2	15.92	602	9.14	356	1.47069777	1.000329527	0.032952688	0.999670582	15372.19	15353.7323
3	6.78	958	31.31	808	2.219101194	1.0007505	0.075049963	0.999250063	9904.6675	9901.404721

$$\theta = tan^{-1}\left(\frac{\Delta z}{\Delta x}\right)$$

θ *is the slope angle*

$$S = \frac{A_{Slope}}{A_{Proj}} = \sqrt{1+\left(\frac{\Delta z}{\Delta x}\right)^2}$$

A_{proj} = projected bird's-eye ROI area

A_{slope} = slope-corrected macroscopic surface area

Δz = coating thickness difference between two regions

Δx = lateral distance between those region
